# Multidimensional family therapy in adolescents with a cannabis use disorder: long-term effects on delinquency in a randomized controlled trial

**DOI:** 10.1186/s13034-018-0248-x

**Published:** 2018-08-17

**Authors:** Thimo M. van der Pol, Vincent Hendriks, Henk Rigter, Moran D. Cohn, Theo A. H. Doreleijers, Lieke van Domburgh, Robert R. J. M. Vermeiren

**Affiliations:** 1 Department of Child and Adolescent Psychiatry, Curium-Leiden University Medical Center, Leiden, The Netherlands; 2 Department of Child and Adolescent Psychiatry, VU University Medical Center, Meibergdreef 5, 1105 AZ Amsterdam, The Netherlands; 3 Intermetzo-Pluryn, Nijmegen, The Netherlands

**Keywords:** Delinquency, Criminality, Adolescents, Cannabis use disorder, Multidimensional family therapy, Cognitive behavioral therapy, Randomized controlled trial

## Abstract

**Background:**

Substance use and delinquency are considered to be mutual risk factors. Previous studies have shown that multidimensional family therapy (MDFT) is effective in tackling both conditions on the short term. The current study examines the long-term effects of MDFT on criminal offending.

**Methods:**

109 adolescents with cannabis use disorder and comorbid problem behavior were randomly assigned to either MDFT or cognitive behavioral therapy (CBT). Police arrest data were collected for 6 years: 3 years prior to and 3 years after treatment entry. Using survival analysis and repeated measure General Linear Models (rmGLM), the two treatment groups were compared on number of arrests, type of offence, and severity of offence. Moderator analyses looking at age, disruptive behavior disorders, history of crimes, family functioning, and (severe) cannabis use were conducted (rmGLM).

**Results:**

While police arrest rates increased in the 3 years before treatment, the rates decreased substantially after the start of both treatments. No differences were found between the treatment groups with respect to either time to first offence from the start of the treatment or changes in frequency or severity of offending over time. A treatment effect trend favoring MDFT was found for property offending in the subgroup of adolescents with high baseline-severity of cannabis use.

**Conclusions:**

Across a follow-up period of 3 years, MDFT and CBT were similarly effective in reducing delinquency in adolescents with a cannabis use disorder.

*Trial registration* ISRCTN51014277, Registered 17 March 2010—Retrospectively registered, http://www.isrctn.com/ISRCTN51014277

## Background

In adolescence, substance use disorder (SUD) is often part of multi-problem behavior, characterized by comorbid delinquency, truancy, and (other) psychopathology [1, 2]. The co-occurrence of SUD and delinquency is particularly common [3–6]. While substance use (disorder) is a risk factor for criminal offending [7]. Conversely, delinquency is a risk factor for the development of SUD [8]. Because of the interrelatedness between the two conditions, clinicians and researchers have investigated treatments which aim to target both substance use disorders and delinquency.

Treatments addressing multiple behavioral problems of youth are likely to be more effective on any therapy outcome than treatments targeting a single problem [9, 10]. Of the individual (adolescent-focused) treatments, cognitive behavioural therapy (CBT) has been examined most often. Systematic reviews and meta-analyses have revealed the potential of both treatments to reduce substance use (disorder) and delinquency simultaneously [11–13]. Family therapies and cognitive behavioral therapy (CBT) have been examined most thoroughly in this respect. The meta-analysis of Baldwin [14] reports a slightly larger effect for family therapies like multidimensional family therapy (MDFT) compared to other therapies (including CBT) on delinquency and substance use reduction. In sum, looking at the literature, both CBT and MDFT seem to be able to address multiple-problem behaviors, like SUD and delinquency [11].

Crucial for the success of treatments in decreasing criminal offending is the capacity to target specific risk factors associated with (the development of) delinquency of the youth [15]. The Risk Need Responsivity Model (RNR) states that besides leveling the intensity of treatment to the risk of re-offending (the risk principle), it is important to assess the criminogenic needs of an offender and to match the cognitive ability, motivation and learning style of the offender with the treatment [9, 16, 17]. Several studies revealed good results for both MDFT and CBT [18], sometimes favoring MDFT [19–22], in the reduction of short-term criminal behavior. To examine which treatment works best for which adolescent in decreasing long-term criminal offending, comparing MDFT and CBT can generate important insights.

In criminological research, both self-reported criminality data and official crime records are used to identify and monitor delinquency. While the use of self-report data is common and accepted as a valid measure of crime reduction, reductions of official crime levels are often used as markers of effectiveness of forensic interventions by policy makers in order to adapt or change policies. Self-report data may be biased, with respondents holding back on confessing all transgressions of the law. On the other hand, self-report may invite respondents to also report criminal offences that went unnoticed to police and justice authorities. Database crime records may be more objective, but are often far from complete [23]. In the studies cited, the effect of treatment on delinquency was assessed from adolescents’ self-report of criminal offences committed, with exception of Dakof et al. [19], who collected crime data from registries to complement the self-reports from the studied participants. Therefore, investigating a longer follow up period of official police arrest data should reveal complementary information about possible desistence or durability of criminal offending.

The present study extends a previous randomized controlled trial conducted by Hendriks et al. [18] on the potential of MDFT and CBT to decrease the rate of cannabis use disorder (CUD) in adolescents. In the current study, the long-term effects on delinquency of the two treatments are investigated by analyzing the police arrest records of the participants. The first aim was to evaluate the development of criminal offending for the studied adolescents with a CUD, and to compare the long-term effectiveness of MDFT and CBT in reducing delinquency. The second aim was to investigate whether baseline characteristics of the adolescent differentially predicted treatment effect—reduction of registered arrests—in MDFT and CBT. We hypothesized that both treatments would reduce criminal offending while subgroups with high prevalence of CD/ODD, or high-severity CUD/SUD, would benefit more from MDFT than from CBT.

## Methods

### Sample

Table 1 lists several demographic characteristics of the population. As established earlier, these characteristics (except for drug offences) did not differ between the two treatment groups [18]. The study included 109 Dutch adolescents, mostly boys (80%), between 13 and 18 years of age (mean age 16.8 years [SD 1.3]). The majority (72%) was of Dutch or another Western ethnicity (Table 1). All participants were diagnosed with DSM-IV cannabis abuse or dependence and 66% had a criminal arrest history (one or multiple arrests) at the start of treatment. The sample of this study was enrolled in a Dutch randomized controlled trial, which was conducted as part of a transnational trial (Germany, France, Belgium, Switzerland, and the Netherlands) comparing the effectiveness of MDFT and treatment as usual (TAU) in adolescents with a CUD, i.e. the INCANT study [24]. Treatment as usual was individual psychotherapy, which was CBT in the Netherlands. The trial in The Netherlands was approved by the medical-ethical committee for research in mental health care settings of The Netherlands (METiGG; registration nr. 5238). Per adolescent at least one (step)parent or legal guardian participated in the trial. All adolescents and parents provided written informed consent to join the study. Most adolescents (73%) were referred to the study’s treatment centres by mental health and youth care professionals from other treatment facilities; 19% were referred by Justice authorities, usually a youth probation officer. 8% were self-referred or referred by family or other acquaintances [25]. Adolescents were barred from the study if they were currently psychotic (DSM-IV), suicidal or mentally retarded (clinical judgment), needed inpatient or opioid substitution treatment (clinical judgment), lived outside the catchment area of the treatment centre, or insufficiently understood the Dutch language [18].

**Table 1:** Baseline characteristics of study sample

	MDFT (n = 55) mean (SD)/%	CBT (n = 54) mean (SD)/%	Total sample (n = 109) mean (SD)/%
Demographic background			
Age (range 13–18 years) (years)	16.6 (1.3)	16.9 (1.2)	16.8 (1.3)
Gender male (%)	80.0	79.6	79.8
Ethnicity Dutch/western (%)	72.7	70.4	71.6
Delinquency^a^			
Total offences (%)	72.7	59.3	66.1
Misdemeanor offences (%)	10.9	11.1	11.0
Drug offences^b^ (%)	0.0	7.4	3.7
Vandalism (%)	23.6	18.5	21.1
Property offences (%)	45.5	42.6	44.0
Violent offences^c^ (%)	45.5	50.0	47.7
Sexual offences (%)	1.8	0.0	0.9
(Attempted) manslaughter (%)	5.5	1.9	3.7
Arson (%)	0.0	1.9	0.9
(Attempted) murder (%)	0.0	0.0	0.0
Ever in prison (%)	42.6	37.0	39.8
Sum severity score^d^ (SD)	17.4 (19.9)	15.4 (16.9)	16.4 (18.4)
DSM-IV diagnosis (past year)			
Conduct disorder (CD) (%)	34.8	22.9	28.7
Oppositional deviant disorder (ODD) (%)	19.6	14.9	17.2
CD and/or ODD (%)	43.5	31.9	37.6

*MDFT* multidimensional family therapy, *CBT* cognitive behavioral therapy, *SD* standard deviation, *n* number

^a^Offences committed before start of the treatment, as inferred from police arrest data

^b^Significant difference p < 0.01, all other measures no significant differences

^c^Moderate, sizable and serious violent offences are included

^d^Frequency of offences × severity score of offence using the BOOG-scale

### Treatment sites

Treatment sites were Parnassia Brijder (Mistral unit) and De Jutters (Palmhuis unit), both serving the city of The Hague and the surrounding region. Parnassia Brijder offers outpatient, inpatient, and rehabilitation-oriented addiction care; the Mistral unit is specialized in outpatient care for youths. De Jutters is a child and adolescent treatment agency; Palmhuis offers outpatient care to youths with a variety of problem behaviour, including addiction and delinquency.

### Treatments

MDFT was delivered by 12 MDFT certified therapists who were part of one of two adjoined teams, with two therapists additionally serving as team supervisors. Manualized MDFT offered sessions scheduled twice a week on average. Sessions were held in roughly equal proportion with the adolescent, parent(s), and family (adolescent + parent = family session), respectively, and furthermore with representatives of other systems (school, work, friends, agencies). Sessions could take place at the office, but also at the family’s home or any other convenient location. Scheduling sessions was not limited to regular office hours. The two MDFT teams met once a week to discuss cases and issues.

The comparison treatment (the treatment as usual) was CBT. CBT was carried out by the same treatment centers offering MDFT, but procedurally separated to avoid ‘contamination’ of therapists and participants between the experimental and control conditions. The 14 CBT trained therapists worked as a team, supervised by an outside expert. CBT included sessions with the adolescent, but not with parents and families, held on average once every 2 weeks. Procedures about assessments, urine testing, medication, consultation of other professionals were the same as for MDFT. CBT, like MDFT, started out with treatment engagement interventions and offered psycho-education: informing the adolescent about drugs, delinquency, the maturing of the brain, situations eliciting problem behaviour, the influence of peers, and the importance of protective factors. Sessions were held in the office of the therapist.

### Procedures

In the trial, the recruited adolescents (N = 109) were randomly assigned to outpatient MDFT (N = 55) or outpatient CBT (N = 54)). Independent certified assessors—MSc and PhD students from the University of Miami-rated MDFT treatment integrity applying the validated MDFT Treatment Adherence Scale to video recordings of mid-treatment family sessions [26]. This scale could not be applied to CBT, as there were no family sessions in this treatment condition. In the CBT condition treatment integrity was monitored through training and supervising therapists in CBT [18, 26]. Both treatments had a planned duration of 6 months. The last follow-up assessment was scheduled at 12 months after baseline (see: [18] for an extensive description of the trial). With permission of the WODC—the research institute of the Ministry of Security and Justice of the Netherlands—we retrieved the police arrest records from the National Police Information Services database (IPOL) for all 109 adolescents for a time period of 6 years: 3 years preceding treatment-entry in the trial and 3 years after the start of the treatment. One MDFT case and 7 CBT cases did not start with the assigned treatment (treatment drop-out). As for study drop-out, there was no loss of cases, in any follow-up year.

**Fig. 1:**
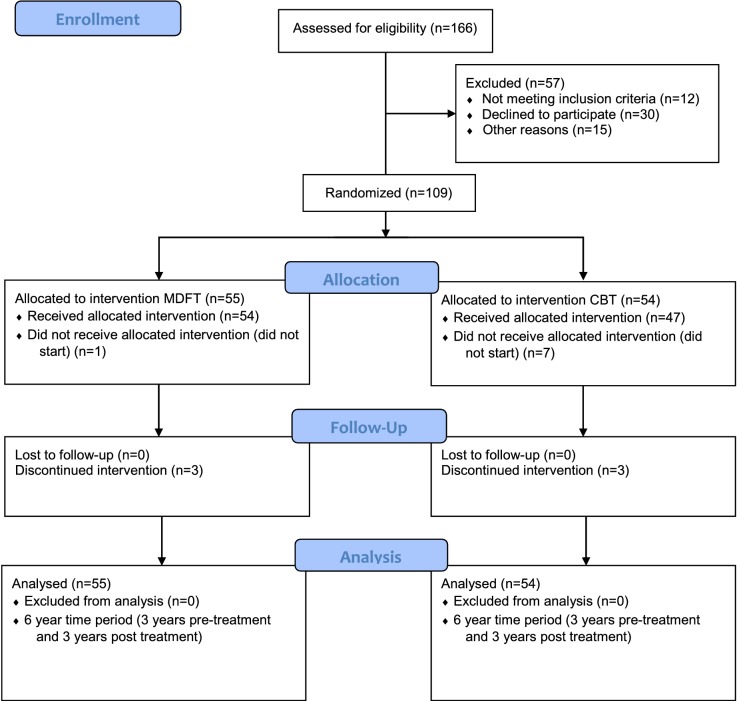
Study flow chart

Figure 1 shows the flow diagram for the study reported here.

### Assessments: criminal offences

Offences were classified and severity was scored using the Dutch BOOG scale [27]. The Boog scale classifies specific law codes into a 12-degree severity index as follows: (1) misdemeanor; (2) drug offence; (3) vandalism; (4) property offence; (5–7) moderate, sizable or serious violent offence; (8) sexual offence; (9) pedosexual offence; (10) (attempted) manslaughter; (11) arson; and (12) (attempted) murder. Three categories were formed for analytical purposes: total offences (all classifications of the BOOG scale, 1–12); violent offences (classifications 5–12 of the BOOG scale); and property offences (classification 4 of the BOOG scale).

### Assessments: cannabis use and mental health

Research assistants who were independent from the treatment staff carried out the assessments. The National Institute of Mental Health Diagnostic Interview Schedule for Children Version IV [NIMH DISC-IV; 28] was administered to determine the presence of a conduct disorder (CD) and oppositional defiant disorder (ODD) over the past year. The prevalence of these two disorders (Table 1) did not differ between the two treatment groups, nor did the prevalence of any other DSM-IV disorder [18].

Family functioning was assessed, using the Dutch version of the Family Environment Scale subscales Conflict (range 0–11) and Cohesion (range 0–11) [FES; 29–31]. Cannabis consumption was measured with the Timeline Follow-Back [TLFB; 32], a calendar method to collect information on the adolescent’s consumption of cannabis in the 90 days preceding each assessment. Adolescents were considered to be low-severity cannabis users if they took cannabis on fewer than 65 days (the baseline median value in the trial) and high-severity users if they consumed the drug on 65 or more days. CUD (DSM-IV) at baseline was established with the Adolescent Diagnostic Interview [ADI-Light; 33], and the Personal Experiences Inventory subscale Personal Involvement with Chemicals (range 0–87) [PEI; 34] was used to determine the adolescents’ level of psychological involvement with substances.

### Statistical analyses

Analyses were run using SPSSv21.0. The adolescent’s first day of treatment was used to mark the three pre-treatment years and the 3 years following treatment entry. First, Kaplan–Meier survival analyses were carried out to examine how long it took for treated adolescents to be (re)arrested by the police, in which potential censoring was taken into account. Pairwise comparisons were made to identify between-group differences (MDFT vs. CBT), using the Log rank statistic. We examined group differences in police arrest and re-arrest incidence, number of offences at issue, and the type and severity of these offences across 6 years (the 3 years before treatment entry, and the 3 years after the start of treatment). The data for the 3 years before and the 3 years after treatment entry, respectively, were analyzed with separate repeated measure General Linear Models (rmGLM) for frequency of: total offences, severity of offences, and type (property and violent offences). We assessed the three pre-treatment years for each year separately, and we did the same for the three consecutive years following the start of the treatment. The time interval chunks were analyzed as a within-subject variable, and treatment as a between-subjects variable.

Moderator analyses were performed to evaluate second-order interactions: age (both continuous and categorical: 13–16 versus 17–18), disruptive behavior disorder status (CD and ODD), history of crimes, family functioning, severe cannabis use, and severe psychological involvement with substance use. To account for any violation of sphericity, we applied Huynh–Feldt-corrected estimates if ∑ ≥ 0.75, and Greenhouse–Geisser correction if ∑ < 0.75 in rmGLM analyses [35].

## Results

### Time to first registered offence

**Fig. 2:**
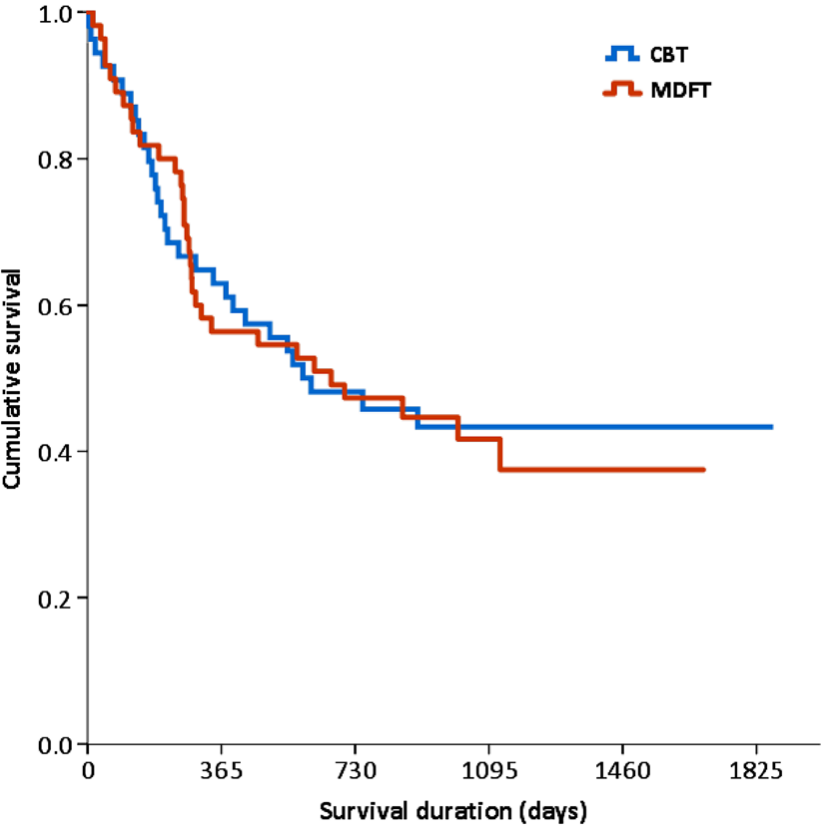
Kaplan-Meier survival curves, showing the duration until first registered police arrest after the start of treatment with MDFT or CBT. *MDFT* multidimensional family therapy, *CBT* cognitive behavioural therapy

Kaplan–Meier survival curve analysis (Fig. 2) yielded no difference between MDFT and CBT (category: total offence) in time to first registered arrest since the start of treatment (log rank test $\upchi_{{1,\text{N} = 109}}^{2}$ = 0.02, p = 0.89).

### Change in frequency over time: total number of offences and the severity of offences

**Fig. 3:**
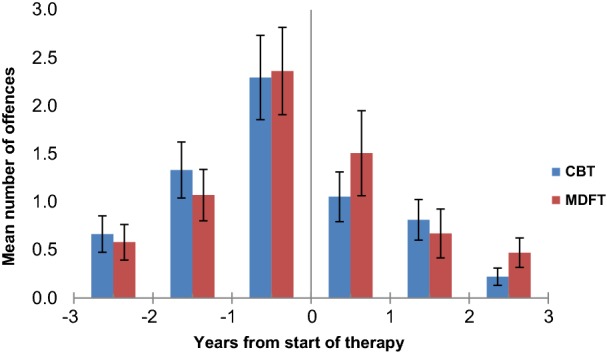
Mean number of total offences (all offences together) per year from the start of CBT and MDFT treatment. *CBT* cognitive behavioural therapy, *MDFT* multidimensional family therapy. Bars: standard deviation

Figure 3 depicts, the total number of police-arrest offences increased in the pre-treatment years and decreased thereafter. For the pre-treatment period, rmGLM analyses showed that the total offences score rose linearly before treatment was initiated in both groups, in terms of offence frequency (time: Huynh–Feldt F_1.7,178.5_ = 16.9, p < 0.001, η^2^ = 0.14; linear F_1,107_ = 32.1, p < 0.001, η^2^ = 0.23), and offence severity (time: Huynh–Feldt F_1.6,175.6_ = 14.1, p < 0.001, η^2^ = 0.12; linear F_1,107_ = 29.5, p < 0.001, η^2^ = 0.22).

From the treatment episode onwards, the number of total offences and the severity of offences dropped to almost zero level (frequency of total offending; time: Greenhouse–Geisser F_2.1,223.6_ = 17.3, p < 0.001, η^2^ = 0.14; severity of offending; time: F_2.0,219.2_ = 14.0, p < 0.001, η^2^ = 0.12) The decrease was linear across the three post-treatment years (total offences: F_1,107_ = 39.5, p < 0.001, η^2^ = 0.27; severity: F_1,107_ = 36.4, p < 0.001, η^2^ = 0.25). The two treatment groups did not differ on these measures (total offences: F_1,107_ = 0.3, p = 0.56, η^2^ = 0; severity: F_1,107_ = 0.4, p = 0.54, η^2^ = 0). There was no significant interaction between treatment and time (total offences): F_2.1,223.6_ = 0.4, p = 0.70, η^2^ = 0; severity: F_2.0,219.2_ = 0.7, p = 0.49, η^2^ = 0.01). Thus, treatment type did not significantly affect changes in offending for the total number of offences or severity over time after the start of treatment. Post-hoc analysis, including offence frequency and severity as covariates, respectively, did not alter our findings.

### Change in frequency over time: violent offences and property offences

#### Before treatment

For police-arrest registered violent offences, the same pattern of increase of pre-treatment arrests was seen in both groups (time: Huynh–Feldt F_1.8,195.0_ = 8.1, p = 0.001, η^2^ = 0.07; linear F_1,107_ = 18.7, p < 0.001, η^2^ = 0.15), without between-subjects (all p ≥ 0.57) or interaction effects (all p ≥ 0.20). For property offences, a similar linear increase in pre-treatment arrest rates was found (time: Huynh–Feldt F_1.7,178.2_ = 7.8, p = 0.001, η^2^ = 0.07; linear F_1,107_ = 15.0, p < 0.001, η^2^ = 0.12).

#### After treatment entry

**Fig. 4:**
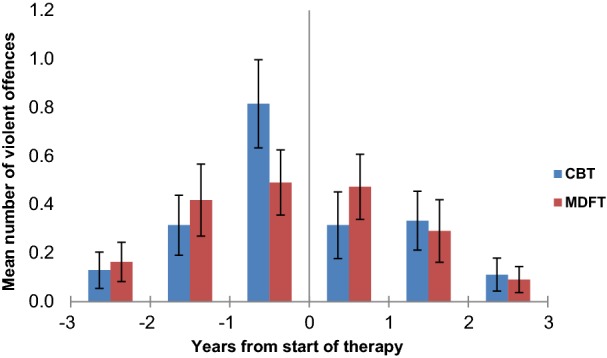
Mean number of violent offences per year from the start of CBT and MDFT treatment. *CBT* cognitive behavioural therapy, *MDFT* multidimensional family therapy. Bars: standard deviation

**Fig. 5:**
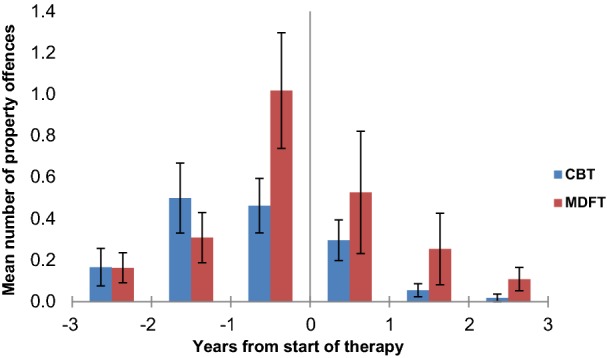
Mean number of property offences per year from the start of CBT and MDFT treatment. *CBT* cognitive behavioural therapy, *MDFT* multidimensional family therapy. Bars: standard deviation

In the three years after treatment entry, the police-arrest rate of violent offences dropped linearly and steeply (Huynh–Feldt; linear F_1,107_ = 19.5, p < 0.0001, η^2^ = 0.15). The same was true of the rate of property offences (Greenhouse–Geisser; linear F_1,107_ = 23,6, p < 0.0001, η^2^ = 0.18). There was no main effect of treatment group and of treatment group by time interaction for violent offence frequency (p > 0.54). With respect to property offending, there was a statistical trend towards a main effect of treatment group, with slightly higher model intercepts in the MDFT group compared to CBT (F_1,107_ = 3.4, p = 0.07, η^2^ = 0.03; MDFT, 1.9 (SD 4.0) vs. CBT, 0.8 (SD 1.5), t_69.4_ = 1.8, p = 0.07). However, there was no treatment group by time interaction, i.e. treatment groups did not differ significantly with respect to the decrease in property offending (p = 0.84). See Fig. 4 (violent offences) and Fig. 5 (property offences).

### Baseline predictors of differential treatment effect

Second-order interaction analyses were carried out to assess if MDFT and CBT differed in reducing police arrest rates when considering baseline characteristics, i.e., age, the presence of conduct disorder or oppositional defiance disorder, crime history, family functioning. All these variables had no effect on crime offending measures in any of the two groups (all p > 0.16).

Baseline severity of cannabis use did not affect treatment response on any measure (all p > 0.20), except for a trend-level three-way interaction with respect to property offending (time*treatment*cannabis use: F_1.7,184.8_ = 3.1, p = 0.056, η^2^ = 0.028). While there was no differential treatment effect in low cannabis using youths (time*treatment p = 0.48), there was a trend towards a steeper decrease in property offending in the MDFT group than in the CBT group in youths with severe cannabis use at baseline (time*treatment F_1.2,64.8_ = 3.5, p = 0.056, η^2^ = 0.06), accompanied by a trend towards a main effect of treatment group (F_1,52_ = 3.8, p = 0.057, η^2^ = 0.07). Inspection of the data indicated that this finding seemed mainly driven by a higher initial level of property offending in the MDFT group compared to the CBT group in high cannabis-using youths (MDFT: 1.6, SD 2.6 vs. CBT: 0.4, SD 0.9), with no differences after treatment (MDFT vs. CBT year 1: 0.2, SD 0.5 vs. 0.2, SD 0.5; year 2: 0.1, SD 0.6 vs. 0.0, SD 0.2; year 3: 0.1, SD 0.4, CBT 0.0, SD 0.0).

## Discussion

The purpose of this study was to evaluate the long-term impact of treatment on the course of delinquency and to compare the effect of MDFT and CBT on registered police arrest of adolescents with a cannabis use disorder. Additionally, we examined if baseline characteristics of the adolescents predicted possible differential treatment outcomes of MDFT and CBT. We assumed that both MDFT and CBT would reduce the rate for criminal offending, with MDFT achieving better results in high-severe subgroups.

Across the 3 years before the therapy began, the rate of criminal offences increased steeply in the study sample. After treatment entry, the rate of criminal offences and the severity of offences declined sharply, to almost zero levels after 3 years. This drop was observed for all our offence measures, and in both groups to the same extent for all offences together, for severity of offences, and for the categories of violent and property offences, respectively.

Moderator analyses indicated that pretreatment patient characteristics (age, disruptive behavior disorder (CD and/or ODD), history of crimes, and family functioning) did not predict differential treatment effect in MDFT and CBT. Only a trend was found in favor of MDFT with respect to decrease in property offences in the subgroup of adolescents with high baseline-severity of cannabis use.

The observed steep decrease of police arrests were found in the most turbulent period of youth, in which the rates for both prevalence and incidence of crime are highest [36]. During this period, the implementation of treatments is considered to be a necessity to prevent possible future persisting criminal activity [37]. One might assume that the initial increase and subsequent decrease in criminal behavior observed in the current study reflect a natural pattern of desistence in late adolescence [38]. This is unlikely, however, as both 13–16 and 17–18-year olds in this study showed a similar strong decrease in criminal activity after the start of the treatment. In addition, it is unlikely that some general trend among all youth in the Netherlands could explain the marked drops in offending measures that were noted in the present study, because for the years covered by our study, national statistics in the Netherlands showed no corresponding decline in arrest rates for all delinquent adolescents in the general population [39].

Contrary to the findings of previous studies that investigated externalizing problem behavior [40], or criminal behavior [19–21, 41], which showed superior results for MDFT, no significant differences between MDFT and CBT were found in the current study. A potential reason could be the use of official crime records, which have a high “dark number” (only detected crimes are recorded), which underrate the actual criminal activity of an adolescent, creating possible bias [23, 42]. The possible impact of treatments on criminal behavior could therefore be underestimated.

Former studies looking at cannabis use [18, 43], criminal behavior [19–21], and a recent meta-analysis of Van der Pol et al. [22] analyzing multiple outcome measures, found indications of the existence of the “severity gradient”—the higher effectiveness of MDFT compared to CBT and other treatments in severe cannabis/substance using adolescents. Therefore, it could be expected that MDFT, would yield better results in specific high-risk groups. The results in this study contrast this hypothesis. A possible explanation could be the rather small size of the treatment groups (total N = 109; MDFT = 55, CBT = 54), for conducting moderator analyses (i.e. the study was relatively underpowered to detect small effect size differences). A recent study that was conducted [22], investigating self-report criminal behavior for a larger group of 169 adolescents, support this possible explanation, because indications for the “severity gradient” were reported in this study.

One of the assets of the present study was its long time-span (6 years), both before and after treatment, presenting a comprehensive overview of the development of criminal behavior across the major part of adolescence. Our data provide the urgently needed across-years perspective, which was lacking in previous studies. Another strength of this study is the use of a randomized control trial design, which is considered to be the most robust design and best equipped to handle threats to a study’s internal validity [44, 45]. Furthermore, this study is the first in Europe comparing adolescents receiving MDFT or CBT with respect to official crime records, providing an addition to the evidence base stemming from the United States. A final asset is the low study drop-out rate, both in our earlier study focusing on cannabis use outcomes [46] and in the present study, with 0% study drop-out.

Some limitations must be mentioned. The sample (109 adolescents) was rather small, although big enough to demonstrate treatment effects in another investigation [18]. Our self-report study included a larger sample: not only the Dutch but also the Swiss INCANT cohort. Of all INCANT cohorts (from five countries), the Dutch one was possibly among the least impaired, with relatively low levels of cannabis dependence and alcohol use disorder [46]. As discussed, impairment level (severity of cannabis [ab]use) has been found to modify treatment responses. A limitation, too, was the absence of a third treatment group, viz., adolescents receiving no treatment at all. We did not include such a group, as withholding youths an effective treatment would have been unethical.

For future research, we suggest to investigate large groups of adolescents, looking at both self-report questionnaires and official crime records longitudinally, to gain a more comprehensive insight for this complex group of adolescents. Furthermore, we suggest further disentanglement of the underlying mechanisms of criminal behavior, which didn’t fit in the scope of this study. For example, different risk profiles (compare adolescents with one or combinations of multiple risk factors) could give more direction for future research and make it possible to further explore the possible differences of effectiveness of evidence based treatments targeting delinquency [7, 47]. Moreover, studying a more persisting group of delinquent adolescents could be beneficial for identifying risk factors and possible outcome measures related with reduction of criminal behavior.

## Conclusions

With trials conducted at American and European sites, using self-report and registry data, it is safe to conclude that both MDFT and CBT are evidence-based treatments not only for substance abusing but also for delinquent adolescents. By not clearly showing that MDFT is superior to CBT in achieving behavioural change, the present study is somewhat at variance with earlier studies, but the ability of both examined treatments to lastingly reduce criminal offending rates to almost zero levels is nevertheless in line with the results of earlier studies. The outcomes of a series of studies, within and outside INCANT, suggest that MDFT and CBT are equally effective in reducing crime rates in mildly impaired adolescents, however defined. MDFT is to be preferred when the impairment, e.g., cannabis (ab)use severity level, is relatively large. The final choice of treatment may be dictated by cost considerations. Although the initial cost of MDFT are higher than CBT. A cost-effectiveness analysis targeting both personal, medical, and social costs of varied adolescent problem behaviours in relation to treatment, for the same population of adolescents featuring in the present study, found MDFT to be slightly more cost-effective than CBT [48].

## Abbreviations

ADI: Adolescent Diagnostic Interview
CBT: cognitive behavioural therapy
CD: conduct disorder
INCANT: International Cannabis Need of Treatment trial
MDFT: multidimensional family therapy
MST: multisystemic therapy
ODD: oppositional defiant disorder
PEI: personal experiences inventory
RCT: randomized controlled trial
rmGLM: repeated measure General Linear Models
SD: standard deviation
TLFB: Timeline Follow-back
